# Inconsistent effects of stochastic resonance on human auditory processing

**DOI:** 10.1038/s41598-020-63332-w

**Published:** 2020-04-14

**Authors:** Katharina S. Rufener, Julian Kauk, Philipp Ruhnau, Stefan Repplinger, Peter Heil, Tino Zaehle

**Affiliations:** 1 Department of Neurology, Otto-von-Guericke University, 39120 Magdeburg, Germany; 2 Center for Behavioral Brain Sciences, 39120 Magdeburg, Germany; 3 OVGU ESF International Graduate School ABINEP International Graduate School on Analysis, Imaging and Modelling of Neuronal and Inflammatory Processes, 39120 Magdeburg, Germany; 4 Department of Systems Physiology of Learning, Leibniz Institute for Neurobiology, 39118 Magdeburg, Germany

**Keywords:** Human behaviour, Auditory system, Sensory processing

## Abstract

It has been demonstrated that, while otherwise detrimental, noise can improve sensory perception under optimal conditions. The mechanism underlying this improvement is stochastic resonance. An inverted U-shaped relationship between noise level and task performance is considered as the signature of stochastic resonance. Previous studies have proposed the existence of stochastic resonance also in the human auditory system. However, the reported beneficial effects of noise are small, based on a small sample, and do not confirm the proposed inverted U-shaped function. Here, we investigated in two separate studies whether stochastic resonance may be present in the human auditory system by applying noise of different levels, either acoustically or electrically via transcranial random noise stimulation, while participants had to detect acoustic stimuli adjusted to their individual hearing threshold. We find no evidence for behaviorally relevant effects of stochastic resonance. Although detection rate for near-threshold acoustic stimuli appears to vary in an inverted U-shaped manner for some subjects, it varies in a U-shaped manner or in other manners for other subjects. Our results show that subjects do not benefit from noise, irrespective of its modality. In conclusion, our results question the existence of stochastic resonance in the human auditory system.

## Introduction

Noise is generally considered detrimental for signal processing. Under certain circumstances, however, noise can be beneficial. It has been demonstrated in a variety of micro- and macro-systems that applying noise to a weak signal can increase the probability that the signal crosses a threshold^1–3^. Given such a threshold, the probability of detecting a subthreshold signal may therefore be higher in the presence of noise at a specific optimal level than in the absence of noise or in the presence of noise of suboptimal and supraoptimal levels. This results in a non-linear relationship between the probability of detecting a weak signal and the noise level. With regard to sensory perception, an inverted U-shaped function between signal and noise is typically considered as the signature of stochastic resonance (SR)^4–7^.

Animal data suggest that the responses of hair cells, auditory-nerve fibers, and brainstem neurons to weak acoustic stimuli are enhanced by low levels of acoustic noise in a manner consistent with stochastic resonance^7–10^. Human electrophysiological data suggest that noise can increase neural synchrony in the human auditory cortex, which, in turn, might lead to improved hearing performance^11^. Furthermore, a few studies claim improvements in perceptual detection thresholds due to the application of low-level noise. This has been reported for normally hearing subjects^9,12^ and for subjects having cochlear and brainstem implants^9^.

However, although this previous research argues for a beneficial effect of noise on auditory perception, to the best of our knowledge, none of the available studies provide robust evidence for SR in the human auditory system, either due to conceptual shortcomings or to a lack of a valid statistical analysis. Firstly, no study shows the expected SR-inherent inverted U-shaped relationship between noise level and performance in humans^9,11,12^. Rather, only one single noise level leads to improved detection while noise levels below or above fail to do so. Secondly, data in favor of SR in the auditory system typically rely on a small number of subjects^9,12,13^, which increases the probability of false-positive results. Thirdly, one study^9^ proposing SR lacks a statistical analysis such that the reported results are of descriptive nature only.

In addition to the critical role of external noise, the amount of internal noise present has also been discussed as a crucial predictor of SR-effects in human perception^2,14–16^. Internal noise includes any kind of non-phase-locked activity in the system at any stage of the signal transmission, from basic physiological properties, for instance noise generated in neurons to higher order cognitive functions such as fluctuation in the subject’s attention. Walsh (2004)^14^ suggested that internal and external noise together determine the overall amount of noise that is added to the weak external signal. Internal noise is typically estimated as the subject’s variability in performance without the application of external noise. In the visual system, a negative correlation between the amount of internal noise and SR has been demonstrated^15^. Accordingly, it has been suggested that the likeliness of SR to occur is reduced if the internal noise itself is already very strong. Adding external noise would then cause masking of the target stimulus^16^. However, to date, no study assessing SR in the auditory domain took the role of internal noise into account.

Here, we applied acoustic noise at different intensities to assess the existence of SR in the auditory system, as manifested by an inverted U-shaped relationship between noise level and detection performance (study 1). In order to investigate whether SR emerges at the level of the auditory cortex, we applied electric white noise at different intensities by means of transcranial random noise stimulation (tRNS, study 2). TRNS is a non-invasive brain stimulation technique that applies weak electrical currents of randomly changing frequencies and amplitudes. Although the precise neurophysiological mechanisms behind tRNS-effects are yet not fully understood, increased cortical excitability in circumscribed areas under the stimulation electrodes has been demonstrated^17^. On the behavioral level, the efficacy of tRNS to increase detection rate for near-threshold stimuli has been shown both in the visual^18^ as well as in the auditory domain^19^.

In both studies, we first analyzed the detection performance at the group level by means of a non-parametric approach. Secondly, to take into account the high inter-individual variability we performed a common “best compared to zero-noise condition” comparison. Thirdly, we fitted a linear and a quadratic function to the individual data in order to compare the fits of these two model functions. Finally, we estimated the amount of internal noise, as reflected by the subjects’ variability in detecting target stimuli in the zero-noise condition, and predicted SR on the basis of this approximation.

## Results

### Study 1: Assessing the effect of acoustic noise on detection rate for near threshold stimuli

**Figure 1:**
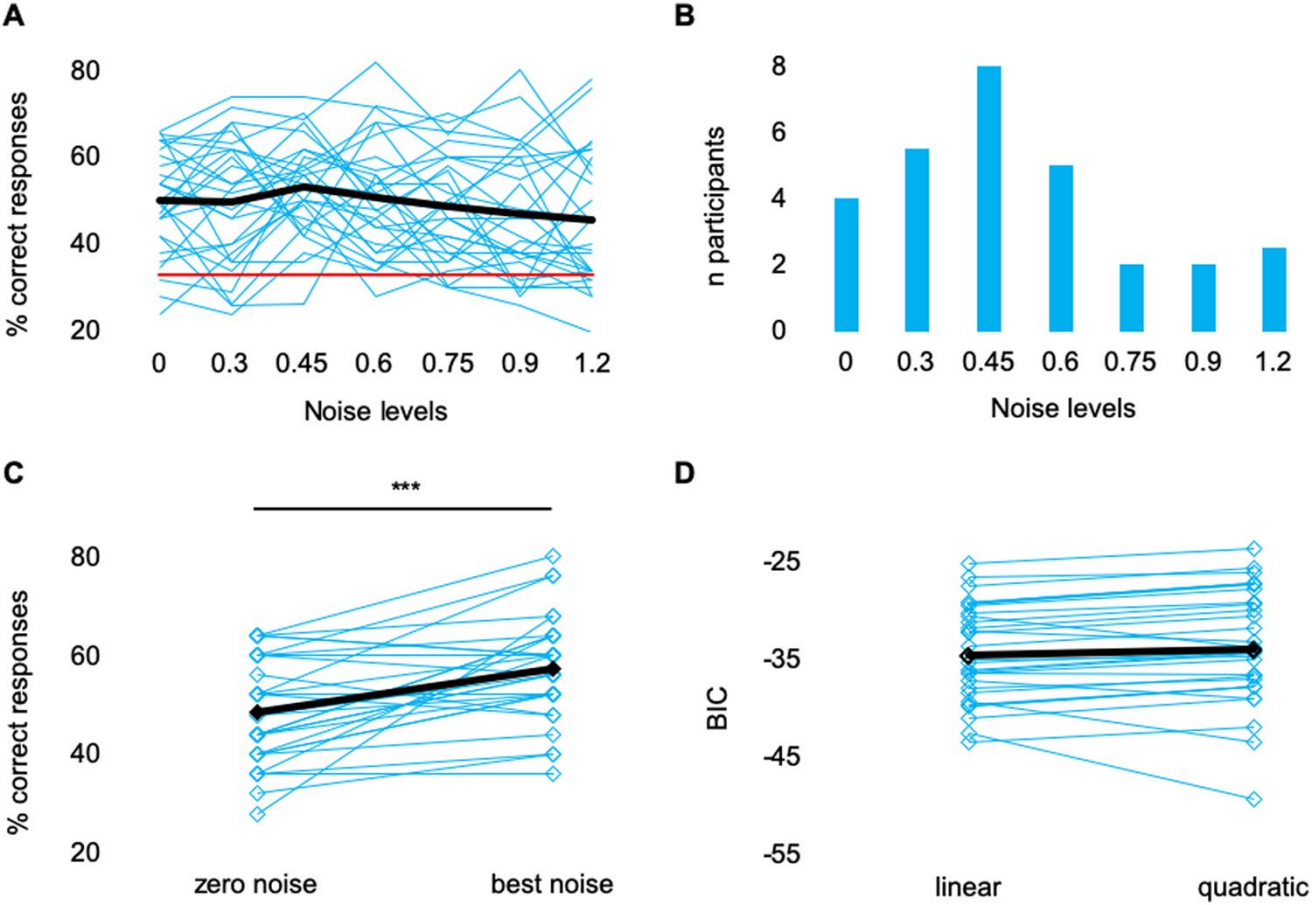
Results of the acoustic stimulation in study 1. (**A**) Behavioral performance depicted for each noise level. Cyan lines indicate the individual participants, the black line the mean performance and the red line the chance level (33% in a 3AFC-task). (**B**) Histogram showing the number of individuals that performed best at each noise level. (**C**) Mean performance (black rectangles) and individual performance (cyan rectangles) in the zero-noise condition and in the best noise condition. Lines connect data from a given subject. Asterisks indicate statistically significant differences. (**D**) Mean BICs (black rectangles) and individual BICs (cyan rectangles) for the linear and the quadratic fit demonstrating that the quadratic fit does not better explain the relationship between detection rate and noise level than the linear fit. Lines connect data from a given subject.

In this study, participants were presented with near-threshold acoustic target stimulus embedded in white noise applied at six different intensity levels (individual detection threshold multiplied by 0.3, 0.45, 0.6, 0.75, 0.9, 1.2). Using a three-interval-three-alternative forced choice (3AFC) task, participants were to indicate in which observation interval the target stimulus was presented. We hypothesize to find 1) an inverted U-shaped relationship between noise level and detection rate and 2) an increased detection rate when the target stimulus was presented embedded in noise compared to when presented in the zero-noise condition. At a group level, the level of the acoustic noise had a small differential effect on the detection probability (Friedman test; chi-square = 12.974, p = 0.043; cf. Fig. 1A). However, post hoc comparisons of the detection probability at each noise level with that in the zero-noise condition did not reveal significant differences (Wilcoxon paired signed-rank tests; all p > 0.05, even if not corrected for multiple comparisons). The detection probability for the noise level of 0.45 was higher than that for all other noise levels (excluding the zero-noise condition). After Bonferroni-Holmes correction, however, none of the differences were significant. In the Bayesian ANOVA, the null model (no performance difference between the noise levels) was favored, but the evidence for the null model was inconclusive (BF 1.3).

Figure 1B shows the distribution of the noise level at which the participants achieved their best performance (“best noise” level) emphasizing the pronounced variability. We compared the performance in the individual best noise condition to the performance in the zero-noise condition (Fig. 1C) (see Methods for details). A significantly increased detection rate was found when noise at the optimal level was applied compare to zero-noise ((Z(28) = −3.668, p < 0.001).

In order to examine whether there may be an inverted U-shaped relation between noise level and detection probability, we compared the goodness-of-fit (quantified by the Bayesian information criterion, BIC) of the quadratic and the linear fit of the participants’ behavioral data. As evident from Fig. 1D, we found no statistically significant difference between the BICs of the linear fit and the quadratic fit (Z(28) = −1.724, p = 0.085). In fact, only six out of 29 subjects showed a superiority of the quadratic fit combined with a < 0, indicating an inverted U-shaped rather than a linear relationship between noise level and task performance. However, with a mean BIC-difference of M = −2.64, SD = 2.31, the evidence against the linear model even in this subgroup was only small.

**Figure 2:**
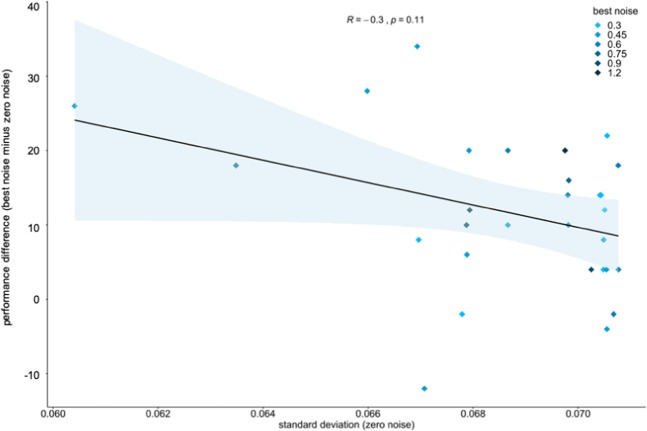
Internal noise as predictor for an SR-effect in study 1. The scatterplot depicts the lack of a statistically significant correlation between the subject’s internal noise, i.e., the variability in the zero-noise condition, and the occurrence of an SR-effect as represented by the difference between the subject’s performance achieved in the best noise condition and in the zero-noise condition. Rectangles depict the different subjects. The best noise condition is color-coded.

Finally, we estimated each subject’s internal noise level, as reflected by the variability in performance in the zero-noise condition. We then used this parameter as a predictor for SR, as quantified by the subject’s performance increase from the zero-noise condition to the best noise condition. Figure 2 shows the lack of a statistically significant relationship between internal noise and SR (Spearman’s rank correlation coefficient, r = 0.3; p = 0.11), indicating that the subjects’ variability in the zero-noise condition does not predict an SR-effect.

### Study 2: Assessing the effect of electrical noise on detection rate for near threshold stimuli

**Figure 3:**
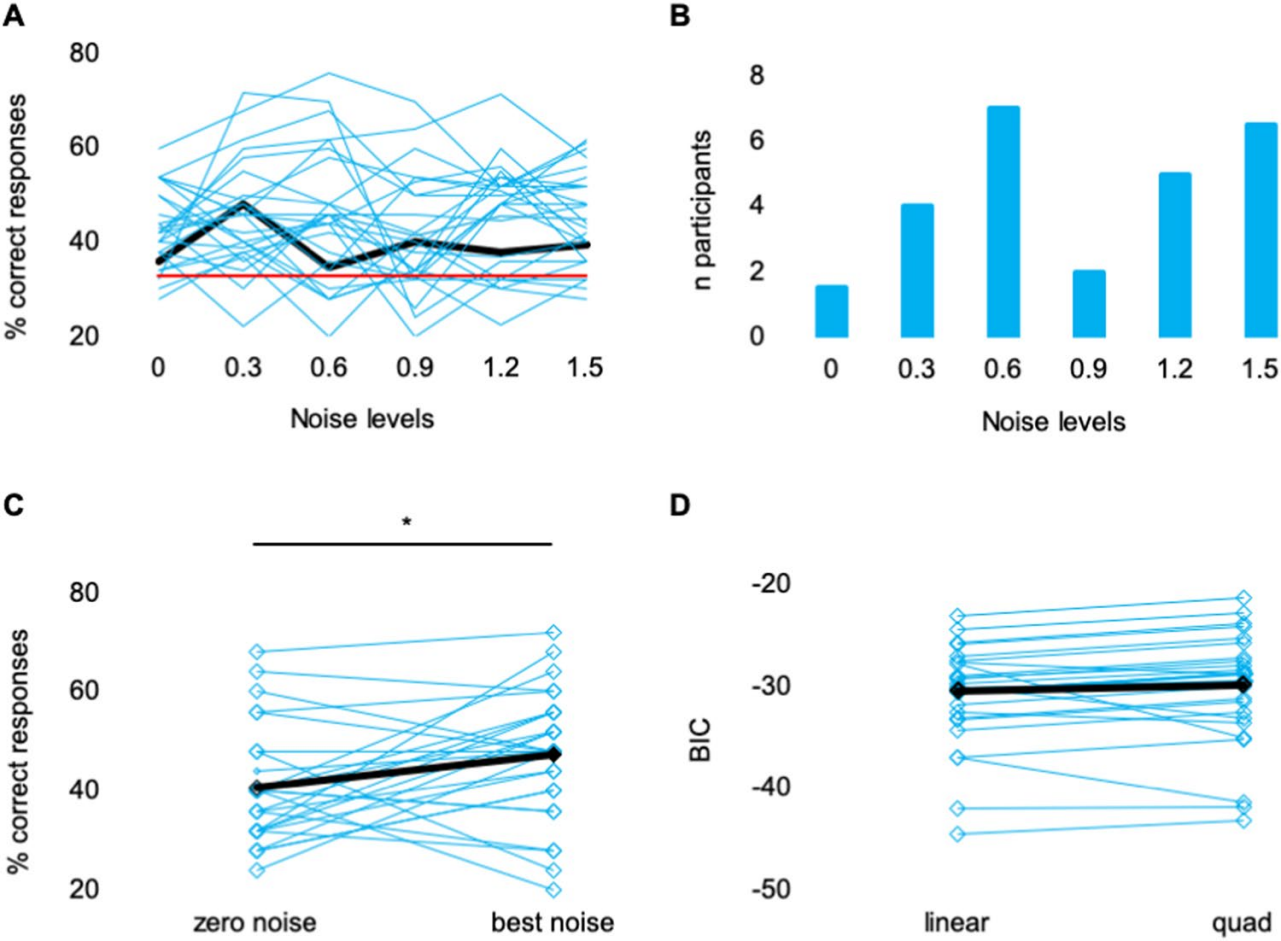
Results of the electrical stimulation in study 2. (**A**) Behavioral performance depicted for each noise level. Cyan lines indicate the individual participants; black line represents the mean performance and red line the chance level (33% in a 3AFC-task). (**B**) Histogram with the number of individuals that performed best at each noise level. (**C**) Mean performance (black rectangles) and individual performance (cyan rectangles) in the zero-noise condition and the best noise condition. Lines connect data from a given subject. Asterisks indicate statistically significant differences. (**D**) Mean BICs (black rectangles) and individual BICs (cyan rectangles) for the linear and the quadratic fit demonstrating that the quadratic fit does not explain the relationship between detection rate and noise level better than the linear fit. Lines connect data from a given subject.

In this study, participants had to detect a near-threshold acoustic target stimulus while tRNS at different intensities (0.3, 0.6, 0.9, 1.2, and 1.5 mA) was administered to the auditory cortex. Using a 3AFC task, participants were to indicate in which observation interval the target stimulus was presented. We hypothesize to find 1) an inverted U-shaped relationship between tRNS intensity and detection rate and 2) an increased detection rate when the target stimulus was presented simultaneously with tRNS compared to when presented without tRNS. At a group level, we found no evidence that the noise level differentially affected the subjects’ detection rates (Friedman test; chi-square = 2.538, p = 0.771; cf. Fig. 3A). This finding was confirmed by the Bayesian JZS ANOVA, which provided strong evidence for the null model (BF 11.9).

Figure 3B indicates the variability in the noise level at which the participants achieved their best performance. We compared the performance in the individual best noise condition to the performance in the zero-noise condition (Fig. 3C) (see Methods for details). A significantly increased detection rate was found when noise at the optimal level was applied compare to zero-noise (Z(25) = −2.041, p = 0.041).

Comparing the BIC for the linear to the BIC for the quadratic model fit (cf. Fig. 3D) showed no superiority for one of the two approaches to explain the relationship between noise level and detection rate (Z(25) = −0.673, p = 0.501). Only five of 26 subjects showed lower BICs in the quadratic fit combined with a < 0, indicating an inverted U-shaped relationship between noise level and detection rate. However, and comparable to the results of study 1, the difference between the two BICs was only moderate even in this subgroup (M = −3.41, SD = 2.56).

**Figure 4:**
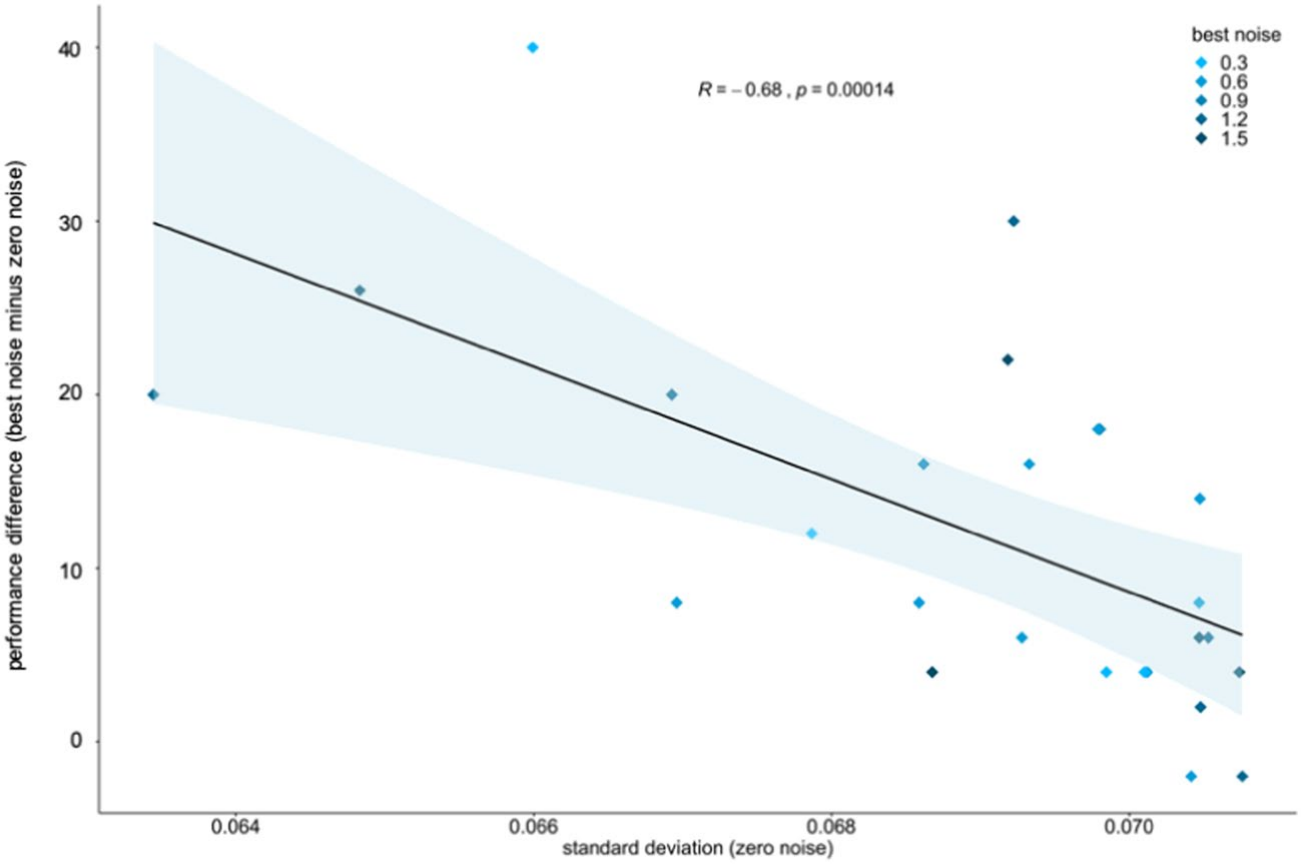
Internal noise as predictor for an SR-effect in study 2. The scatterplot depicts the negative correlation between a subject’s internal noise, i.e. the variability in the zero-noise condition, and the occurrence of an SR-effect as represented by the difference between the subject’s performance achieved in the best noise condition and in the zero-noise condition. Rectangles depict the different subjects. The best noise condition is color-coded.

Finally, we estimated each subject’s internal noise level and used this parameter as a predictor for SR, as quantified by the subject’s performance increase from the best noise condition compared to the zero-noise condition. Figure 4 shows the statistically significant relationship between internal noise and SR (Spearman’s rank correlation coefficient, r = −0.68; p < 0.001), indicating that the subject’s variability in the zero-noise condition predicts an SR-effect.

## Discussion and Conclusion

Here we report the results of two studies investigating the existence of SR in the human auditory system. We applied noise either acoustically (study 1) or electrically by means of tRNS (study 2) to assess detection rates for near-threshold acoustic stimuli as a function of the applied noise level. The results of our studies show that adding low-level acoustic noise has only weak, if any, overall effect on the participants’ detection probability while low-level electrical noise did not yield such an overall effect. However, neither in study 1 nor in study 2 did we find evidence for an inverted U-shaped relationship between noise-level and detection rate. In fact, only a small subsample of participants (six out of twenty-nine in study 1 and five out of twenty-six in study 2) showed the hypothesized superiority of the quadratic inverted U-shaped compared to the linear model fit expected for SR. Moreover, the differences in the BICs for the linear and the quadratic model fits are only small, even when considering only those individuals showing a superiority of the quadratic model fit^20^. Our data thus argue against the general existence of a behaviorally relevant SR-mechanism in the human auditory system.

Previous work on SR has discussed the role of the subjects’ internal noise level (i.e., noise at each stage of signal processing from the cellular to the behavioral level) as a crucial mediator of SR^14–16^. While a high amount of internal noise negatively affects SR because the additional application of external noise then masks the signal, a mild to moderate amount should allow SR^16^. In line with those studies, we found a negative correlation between the subjects’ internal noise and the occurrence of an SR-effect when the external noise consisted of tRNS (study 2). No such effect was present in study 1, where acoustic white noise was applied. This is intriguing because the target stimulus was identical in both studies. However, in study 1 both the target and the noise were acoustic stimuli, and, thus, they already interacted physically. In study 2, the noise consisted of electrical white noise applied by means of tRNS over the auditory cortex. It seems therefore plausible that the amount of internal noise is SR-relevant only if target signal and external noise converge not until the level of the auditory cortex, an argumentation which is in line with previous work on SR in the visual^16^ and the somatosensory system^21^.

What could be other reasons for why we found no consistent evidence for SR in our studies? To address this, it is useful to recall the requirements for SR to occur. In the canonical threshold version of SR, an invariable “hard” threshold is assumed. The sinusoidal signal alone is subthreshold and never crosses that threshold. With weak noise added to the signal, the sum of signal plus noise has an increased probability of periodically exceeding the hard threshold, such that the signal can be detected^22^. Accordingly, if this threshold is variable and fluctuates over time, noise at a given intensity will not always allow the weak periodic signal to pass this threshold. It is unclear, however, whether a hard threshold exists in the auditory system. Heil and Matysiak^23^ performed experiments specifically designed to address the question of whether there is a hard threshold at the perceptual level but found no evidence for the existence of such a threshold (see also^24^). In fact, detection thresholds for tones presented in quiet, and the dependence of threshold on tone duration and envelope, can be well understood without a hard threshold (e.g.,^23,25^). Ward *et al*.^22^ argue that SR might also be observed in the case of a “soft threshold”, i.e., a non-linear transfer function between instantaneous input and output, such as an exponential or logistic function, if sufficiently steep. The phase-locked period histograms of mammalian auditory-nerve fibers to low-frequency tones can be well described by exponential transfer functions relating the instantaneous stimulus pressure to the instantaneous spike rate (e.g.,^26,27^). However, it appears from comparing the data in these studies with the findings of Ward *et al*.^22^ (their Figure 6) that at the low, near-threshold stimulus levels the transfer functions are not steep enough to allow for SR. Later, Greenwood *et al*.^28^ reported that for SR to occur it is important that the signal is located near a transition between a relatively flat and an increasing portion (an ‘edge’) of the transfer function, while the slope of the function, in itself, would not be of great importance. In our view, an exponential function does not have such an edge. In addition, SR will not arise when the transfer function is invertible (as is the case for the exponential and the logistic function), no matter how steep, if the observer uses the Fisher information to decide about the presence of a stimulus^28^. Given the lack of evidence for a “hard” threshold at the perceptual level or for edges in the invertible transfer functions of auditory-nerve fibers, it is perhaps not surprising that we found no clear evidence for SR.

In both of our studies, we found substantial interindividual variability in the noise level yielding the strongest improvement in the participants’ performance (cf. Figs. 1 and 3) although we individually adjusted the level of the target stimulus and the level of the noise stimulus. Such variability has already been demonstrated in studies assessing SR on the single-unit level^1^, in the motor cortex^29^ and in the visual cortex^18^. Different factors such as the baseline neurotransmitter level, synaptic transmission, number of activated postsynaptic receptors, ion concentration, membrane conductance, and effects of previous action potentials, i.e., the refractory period, have been suggested as modulating factors^30^. These individual physiological properties can modulate the subject’s sensory perception and, accordingly, the same amount of (acoustic or electrical) noise can affect hearing performance differentially.

In the same vein, an identical tRNS-intensity can affect individual participants differentially. Physiological factors such as properties of scalp and cranium but also macroscopic differences between participants, e.g. gyrification and grey and white matter volume result in different susceptibility to the electrical stimulation^31,32^. Although the mechanism of action of tRNS is yet not fully clear, potentiation of voltage-gated sodium-channels resulting in long-term potentiation is the most often discussed possibility (e.g.^17,33^. Recently, data from an animal model showed that tRNS can modulate sodium-channels of pyramidal cells according to the SR-typical inverted U-shaped curve with tRNS administered at intermediate intensity leading to the strongest modulation^34^. Thereby, the membrane potential of pyramidal cells in the vicinity of the tRNS-electrodes is more depolarized, which increases the probability that a weak incoming acoustic signal can evoke an action potential and, as a consequence, a subliminal signal passes detection threshold. How intensities from animal studies translate to human research, however, remains an open question that needs to be addressed in the future.

Since the number of studies using tRNS is limited, especially when looking at work in the auditory domain, we have chosen noise-intensity level increments as used in recent studies on SR in the visual system^18,35^. We therefore cannot fully rule out the possibility that applying noise at other intensities might have led to more pronounced effects. We applied tRNS at intensities between 0.3 and 1.5 mA, which is clearly below the individual sensory threshold^36^. This is confirmed by the fact that none of our participants was able to distinguish between the different stimulation intensities or between the zero-noise condition and any other stimulation condition, as reported in the methods section. Whether we need finer noise-intensity level increments applied to auditory cortex to produce SR-like effects remains to be tested in the future.

In the present work, we assessed online effects of tRNS on SR in the human auditory system. Although it has been demonstrated that the application of transcranial alternating current (tACS) for 1 second does not yield measurable after-effects, there is yet no data available on the stability of tRNS-effects on cortical excitability. Therefore, in order to control for such potential carry-over effects, the different tRNS intensities were presented block-wise. In order to allow comparing the results of study 1 (acoustic noise) and study 2 (electrical noise by means of tRNS) we used a block design also in study 1. Previous studies showed that a full randomization of the noise levels is not a prerequisite to evoke SR-effects (e.g.^11,12,15,16,18^). However, our study design does not allow to draw conclusions on potential fluctuations in performance due to, e.g., perceptual learning, attention, and motivation. Future studies using a more extended number of trials per noise level and a fully randomized presentation of the different noise conditions will shed more light on this aspect.

Of note, we found highly significant effects of noise on detection rate when comparing the zero-noise condition with the specific noise condition that yielded the individual’s best performance but the effect vanished when comparing it with the two neighboring noise levels. Accordingly, the results of our studies also demonstrate that comparing the performance in the subjects’ best-noise condition to the zero-noise condition leads to an artificial difference between the two conditions and, thereby, an overestimation of the real noise effect on detection rate. The use of more robust statistical approaches reduces the probability for false-positive findings. Such approaches include 1) excluding the best-noise condition but comparing the performance at adjacent noise levels to zero-noise, 2) analyzing the overall effect of noise on detection rate by means of a general linear model, and 3) examining whether an inverted U-shaped relationship between noise-level and detection rate exists by comparing the BICs of quadratic and linear fits.

In the present studies, we used a 3AFC task to measure the subject’s performance in detecting acoustic near-threshold stimuli embedded in white noise. Accordingly, SR of type E (energy) was assessed since adding the energy of the noise to the energy of the near-threshold signal was hypothesized to elevated the signal + noise stimulus over threshold. In addition to type E, Moss^7^ postulated a type I (information) SR, which is characterized by increased ability to differentiate subliminal information embedded in supra-threshold stimuli. Thus, future studies will shed more light on whether type I-SR is evident in the human auditory system and, if so, to which amount external noise can improve discrimination rate.

In sum, aiming to investigate SR in the human auditory system we assessed detection rates for near-threshold acoustic stimuli embedded in acoustic or electric noise. The present data provide no evidence for a behaviorally relevant SR-typical inverted U-shaped relationship between detection rate and noise-level, independent of the modality in which noise was applied. Further work is needed to gain a comprehensive understanding of SR in human perception and to establish approaches that would cause behaviorally relevant modulations of the (altered) inherent signal to noise ratio in the human auditory system.

## Material and Methods

### Participants

Fifty native German-speaking subjects in the age range of 19–35 years were recruited via advertisement at the University of Magdeburg. Twenty-nine subjects participated in study 1 (acoustic noise, mean age: 23.7 years, standard deviation: 3.6 years, eighteen females) and twenty-six in study 2 (electrical noise, mean age: 24.5 years, standard deviation: 3.9 years, sixteen females). Five subjects participated in both studies. None of the subjects included in studies 1 and 2 reported any neurological or auditory pathology. In addition, subjects who participated in study 2 fulfilled the physiological prerequisites for transcranial electrical stimulation^37^. All participants gave written informed consent in accordance with the Declaration of Helsinki. The ethics committee of the Medical Faculty of the Otto-von-Guericke University of Magdeburg approved the protocols (approval number 90/17).

### General experimental design

All experiments were run in an acoustically shielded booth (Industrial Acoustics, Company, Niederkrüchten, Germany). Subjects were seated in a comfortable chair in front of a 19 inch LCD screen (vertical refresh rate 60 Hz) at a distance of approximately 60 cm. Stimuli were generated in MATLAB R2010a (The MathWorks, Natick, MA, USA) and were presented diotically using circumaural headphones (Sennheiser HDA 200) by means of the Psychophysics Toolbox extensions^38–40^.

### Determining the individual acoustic target stimulus level

The acoustic target stimulus to be detected in the main experiment in both studies was a pure tone (frequency: 1000 Hz, duration: 500 ms including 10 ms cosine-squared ramps) presented either in quiet or in the presence of a soft background acoustic noise (see below). The level of that tone was set individually for each subject such that it matched each subject’s individual threshold level for detecting that tone in quiet. This threshold level was determined using two consecutive procedures. A first rough estimate of the subject’s threshold level was obtained using a yes / no-detection task combined with a one-up-three-down staircase procedure. Here, a clearly detectable stimulus was presented first, and its level was then adjusted stepwise (fixed step sizes 6 dB, 3 dB, 1.5 dB). The mean of the last six reversals served as the first estimate. The algorithm handling this rough threshold estimation was adopted from Soranzo and Grassi^41^ and yields a threshold level corresponding to a detection probability of 79.4%. Second, this first estimate was used as the initial threshold level fed to an adaptive QUEST procedure^42^. In brief, the QUEST procedure is a Bayesian adaptive psychometric method where the level of the stimulus to be presented in a given trial is based on the subject’s responses in all previous trials. Using a Weibull distribution and the initial estimate of the subject’s threshold (see above), the point of maximum likelihood is then chosen as the best estimate for the threshold, and the next stimulus is presented at that level. Two independent and randomly interleaved QUEST staircase procedures, one starting at 0.8 times (1.94 dB below) and the other starting at 1.2 times (1.58 dB above) the initial threshold level estimate, were performed^42^. Each QUEST staircase procedure consisted of 40 trials, which were presented in a 3AFC-task. The average of the two threshold level estimates from these QUEST staircase procedures was taken as the subject’s individual threshold level for the target stimulus in quiet, which corresponded to a detection probability of 0.5. This part of the experiment took about 40 min.

### Study 1: Effect of acoustic noise on near-threshold acoustic target stimulus detection

In study 1, the target stimulus at threshold level had to be detected either in quiet (zero-noise control condition) or in the presence of acoustic Gaussian white noise (bandwidth: 20 to 14000 Hz, duration: 1000 ms including 10 ms cosine-squared ramps) at six different levels. These levels were obtained by multiplying the noise at the individual threshold level by 0.3, 0.45, 0.6, 0.75, 0.9, and 1.2, yielding noise levels ranging from 10.5 dB below to 1.6 dB above the individual threshold level for the noise. Each subject’s individual threshold level for the noise stimulus was determined using the same two consecutive procedures as described above for the target stimulus.

In the main experiment, participants performed an auditory three-interval-three-alternative forced choice (3AFC) task, where the target stimulus at threshold level was presented randomly in one of three observation intervals, while in each observation interval white noise was presented (except in the zero-noise condition). The observation intervals lasted 1000 ms each (i.e., identical to the duration of the white noise stimulus) and were separated by 500 ms. Each observation interval was cued visually by a red rectangle presented simultaneously on the LCD screen (visual angle 0.9°). The first observation interval was preceded by a period of 2000 ms during which a black cross (visual angle 0.6°) was displayed. After the end of the third observation interval, the black cross reappeared and the subject was requested to select the interval thought to contain the target stimulus by pressing a corresponding button with the dominant hand. This response period lasted 3000 ms, and the next trial started. In the conditions, in which the 500 ms long target tone was presented in noise, the 1000 ms long noise started 250 ms before the onset of the tone and ended 250 ms after the offset of the tone.

In an initial practice block, subjects were familiarized with the task and also received feedback about their performance in each trial. Subsequently, the seven different conditions (one control condition and six noise conditions) were applied in separate blocks. Each block consisted of 50 trials. The order of the blocks was counterbalanced across subjects according to a balanced Latin Square design. Both, the subject and the experimenter were blind to the order of blocks. In the main experiment, subjects received no feedback about their performance. Completion of all measurements took about 120 min. on average per subject.

### Study 2: Effect of electrical noise applied by means of tRNS on near-threshold acoustic target detection

In study 2, the acoustic target stimulus was the same as that in study 1. It had to be detected in quiet but either in the absence (zero-noise control condition) or in the presence of high-frequency electrical noise, with amplitudes of 0.3, 0.6, 0.9, 1.2, and 1.5 mA in different conditions. The electrical noise was applied by means of tRNS (100–640 Hz) using two 5 cm ×7 cm-rubber electrodes placed over T7 and T8 (i.e., over the left and right auditory cortex) according to the 10–20 system for EEG electrode placement and via a battery-driven DC stimulator (NeuroConn, Ilmenau, Germany). Ten20 conductive paste (Weaver and Company, Aurora, USA) was used to attach the electrodes to the scalp and to keep impedances below 10 kOhm. The electrical noise, which was created digitally using Matlab (The Mathworks, Natick, MA, USA), was then converted to an analog signal by a digital-to-analog-converter (NI-6212, National Instruments, Austin, Texas, USA), and then fed into the external signal input of the DC stimulator.

In the main experiment, participants performed the 3AFC-task analogously to study 1. In trials in which electrical noise was applied, tRNS started 250 ms prior to the onset of the first observation interval and ended 250 ms after the offset of the last observation interval. The different tRNS-intensities were applied in separate blocks, each consisting of 50 trials. The order of blocks was counterbalanced according to a balanced Latin Square design. Both, the participant and the experimenter were blind to the order of blocks. On average, the experiment took about 120 min. in total (including preparation of the tRNS-electrodes) and was performed in a single session.

After each block, the subjects were asked whether they had felt any sensation due to the electrical stimulation (i.e., headache, nausea, dizziness, loss of concentration, fatigue, phosphenes, skin irritation, itch, prickle on the scalp, heat, burning) by means of a numeric rating scale (0 = no sensation, 6 = very strong sensation). Statistical analysis of these data by means of separate non-parametric Friedman tests for each sensation did not reveal any significant differences between any of the noise conditions (all p > 0.05).

### Statistical analyses

For both studies, the analysis was equal. Trials with missing responses were excluded from data analysis. None of the participants showed more than 10 missing trials.

First, in order to examine whether there is an overall effect of acoustic or electric noise on the participants’ auditory detection probability on the group level, Friedman-tests with the factor noise level (detection rate at each noise level including the zero-noise control condition) were performed for each study. Wilcoxon paired signed-rank tests were run in order to unravel statistically significant differences in the detection probability at each noise level compared to the zero-noise control condition. Bonferroni correction was applied to correct for multiple comparisons. To verify these results, we used a Bayesian ANOVA invoking the Zellner-Siow’s (JZS) approach with repeated measurements design. For simplicity, noise levels (including zero-noise) were treated as a categorical independent variable, and performance (percent correct) was the dependent variable. JZS ANOVA is a Bayesian method that allows accepting either the null or the working hypothesis. We used R (R package version 0.9.12-4.2) and the anovaBF function from the ‘BayesFactor’ package. This function uses default, non-informative priors^43^ and outputs a Bayes Factor, which quantifies the evidence in favor of one model relative to the evidence in favor of the competing model. By convention, Bayes Factors between 3 and 10 are considered substantial, Bayes Factors> 10 are considered strong evidence in favor of either model^44^.

Secondly, because the noise condition in which the individual participants show best performance might vary due to individual physiological differences^30^, we determined the noise condition yielding the highest detection probability for each subject separately (“best noise” condition) and compared the detection probability in that condition to the detection probability in the zero-noise condition by means of non-parametric Wilcoxon paired signed-rank tests. Therefore, we randomly split the data into two sub-samples (50% of the trials of each noise condition). In sample 1, we determined each individuals best noise condition. In sample 2, we then compared performance in the zero-noise condition with performance in the best noise condition identified in sample 1.

Thirdly, in order to examine whether there is an inverted U-shaped relationship between detection rate *y* and noise level x, considered a signature of SR, we fitted a quadratic function (*y* = *ax*^2^ + *bx* + *c*) to each participant’s data. Here, *x* corresponds to the noise levels (including the zero-noise condition) and the intercept parameter *c* to the performance in the zero-noise condition. As a control, a linear function (*y* = *bx* + *c*) was fitted to data. Similar to the quadratic fit, *x* corresponds to the noise levels and the intercept parameter *c* to the performance in the zero-noise condition. Both, the quadratic and the linear model were fitted to the data of each individual subject and compared using the Bayesian information criterion (BIC = *n* ∙ ln (*SSE* /*n*) + *k* ∙ ln (*n*);^45^, where *SSE* is the sum of the squared errors, *n* is the number of data points (here: number of noise levels), and *k* is the number of free parameters. The SSE from the quadratic model is necessarily less than or equal to that from the linear model, but the number of free parameters is three instead of two. The BIC from the quadratic model can therefore be smaller than, equal to, or larger than that from the linear model. The lower BIC indicates the model which fits the data better. When the difference in the BIC values for the linear and the quadratic fit is <2, the strength of evidence against the linear model is “not worth more than a bare mention”^20^. The model fits were conducted with a maximal number of 1000 iterations using the *lsqcurvefit* function (MATLAB Optimization Toolbox).

Finally, in order to estimate the internal noise, we computed each individual’s variability as reflected by the standard deviation of the performance in the zero-noise condition using a bootstrapping *resampling by replacement* approach (n = 1E6 iterations). Assuming a relationship between the internal noise level and the SR effect, we then run a non-parametric Spearman correlation between this measure of variability and the SR effect, as reflected by performance difference achieved in the best noise condition and in the zero-noise condition.

## Supplementary information

Supplementary information.

## Data Availability

The data sets analyzed in the current work are available from the corresponding author on reasonable request.
