# Supplementary material for: Inconsistent effects of stochastic resonance on human auditory processing
Source: Sci Rep. 2020 Apr 14;10:6419. doi: 10.1038/s41598-020-63332-w (PMC7156366; doi:10.1038/s41598-020-63332-w)
Supplement: Supplementary file 1 — Supplementary information. [file 41598_2020_63332_MOESM1_ESM.docx]

**Inconsistent effects of stochastic resonance on**

**human auditory processing**

Katharina S. Rufener, Julian Kauk, Philipp Ruhnau, Stefan Repplinger, Peter Heil, Tino Zaehle


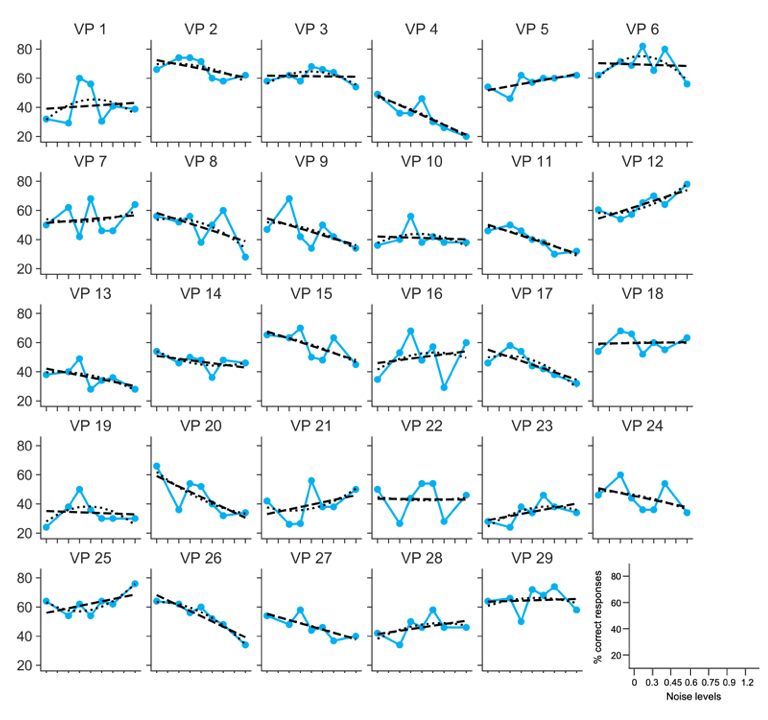


*Supplementary Figure S1: Individual performance separately depicted for all 29 participants of study 1 demonstrating the variability in the modulation of the individual performance. Solid cyan lines represent the individual detection rates, dashed black lines depict the linear fits, and dotted black lines the quadratic fits.*


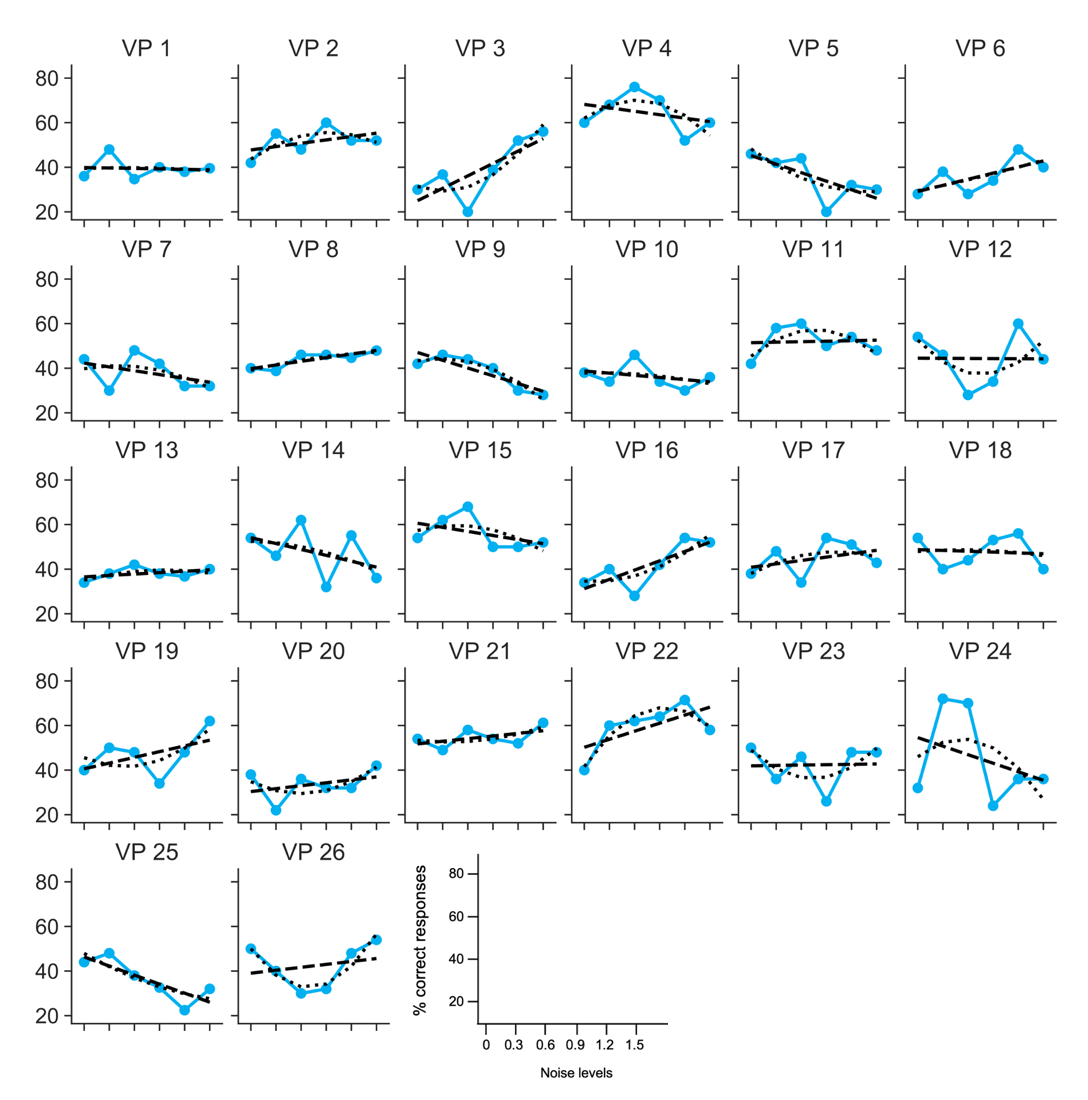


*Supplementary Figure S2: Individual performance separately depicted for all 26 participants of study 2 demonstrating the variability in the modulation of the individual performance. Solid cyan lines represent the individual detection rates, dashed black lines depict the linear fits, and dotted black lines the quadratic fits.*
